# Nanoparticle Vaccine Based on S1 Domain of Porcine Epidemic Diarrhea Virus Elicits Protective Immune Responses in Mice and Pigs

**DOI:** 10.3390/biom16081090

**Published:** 2026-07-25

**Authors:** Pan Tang, Benqiang Li, Enhui Cui, Jie Tao, Jinghua Cheng, Ying Shi, Li Qi, Lilei Lv, Huili Liu

**Affiliations:** 1 Institute of Animal Husbandry and Veterinary Science, Shanghai Academy of Agricultural Sciences, Shanghai 201106, China; tangpan@saas.sh.cn (P.T.); huteng2010@saas.sh.cn (B.L.); taojie@saas.sh.cn (J.T.); zero5cheng@saas.sh.cn (J.C.); shiying@saas.sh.cn (Y.S.); liqi@saas.sh.cn (L.Q.); lvlilei@saas.sh.cn (L.L.); 2 Laboratory Animal Center, Shanghai Jiao Tong University, Shanghai 200240, China; enhuicui@sjtu.edu.cn

**Keywords:** nanoparticle vaccine, spike protein, protective immunity, Ferritin, PEDV

## Abstract

Porcine epidemic diarrhea virus (PEDV) is a major enteric coronavirus that causes severe economic losses to the global swine industry. Current vaccines suffer from weak immunogenicity, insufficient mucosal immunity, and a short duration of protection. Nanoparticle delivery systems have emerged as a promising strategy for next-generation vaccine development. In the present study, we constructed an S1-Ferritin nanoparticle vaccine using the SpyTag-SpyCatcher modular conjugation system. The morphology, particle size, and uniformity of the nanoparticle vaccine were systematically characterized by transmission electron microscopy (TEM) and dynamic light scattering (DLS). After two immunizations, the S-Ferritin nanoparticle vaccine elicited potent humoral and cellular immune responses in both mice and piglets. In piglets, at 2 weeks post booster vaccination, PEDV-specific serum IgG endpoint titers peaked at 1:2560, virus-neutralizing antibody titers reached 1:256 against the JS-2/2015 strain, and serum IFN-γ levels reached 274 pg/mL. All these immunological indicators were significantly higher than those observed in the PEDV-inactivated whole-virus vaccine group. Furthermore, challenge tests showed that the S1-Ferritin nanoparticle vaccine provided complete protection against PEDV infection and significantly reduced the severity of diarrhea and intestinal damage in piglets after challenge. These findings suggest that the S1-Ferritin nanoparticle vaccine constitutes a promising candidate against PEDV infection, though our study is only a preliminary step and subsequent field trials in pigs are still required.

## 1. Introduction

Porcine diarrhea has long been a major disease affecting the pig farming industry, which has caused distress in farmers. It is widely distributed, occurs on a large scale, involves multiple pathogens, and has a complex pathogenesis, resulting in considerable economic losses [1,2]. Among the viral pathogens that cause diarrhea in pigs, PEDV is the most important and destructive [3]. PEDV outbreaks are often acute and are characterized by diarrhea, vomiting, dehydration, and high mortality, posing a serious threat to newborn and suckling piglets [4].

The family Coronaviridae comprises four genera: *Alphacoronavirus*, *Betacoronavirus*, *Gammacoronavirus*, and *Deltacoronavirus*. PEDV is an *Alphacoronavirus* with structural features closely resembling those of other well-documented coronaviruses. According to genetic variation analysis of the S protein sequence, PEDV can be classified into GI, GII, and recombinant S-INDEL strains. Among these, GII can be further divided into GIIa, GIIb, and GIIc. Epidemiological studies have shown that GIIa and GIIb are currently the predominant strains circulating in China [5]. In 2025, the detection rate of the GIIc subtype increased rapidly in China, which warrants close attention [6,7,8]. The continuous evolution of PEDV strains has created significant challenges for disease prevention and control.

PEDV consists of four structural proteins: the spike (S) protein, envelope (E) protein, membrane (M) protein, and nucleocapsid (N) protein. The S protein exists as a trimer on the viral surface and contains two functional subunits, S1 and S2, which perform a variety of biological functions. The S1 domain is a key virulence determinant of PEDV [9]. Amino acid mutations or substitutions within this region can markedly influence viral infectivity, replication efficiency, and pathogenicity in piglets. In addition, the S1 subunit contains multiple dominant neutralizing epitopes, including the core neutralizing epitope (COE) domain and receptor-binding domain (RBD). It serves as the primary antigen responsible for inducing protective immunity and is therefore a critical target for PEDV vaccine development [10].

Vaccination remains the most economical and effective approach for the prevention and control of PEDV. However, currently available commercial vaccines are mainly traditional inactivated vaccines, which provide limited levels of protective antibodies and only short-term immune protection. Live attenuated PEDV vaccines can induce both mucosal and cellular immune responses, but they carry the risk of reversion to virulence [11]. In addition, as a coronavirus, PEDV is highly prone to mutation and recombination, which may lead to reduced vaccine effectiveness. Therefore, the development of new PEDV vaccines is urgently needed. Subunit vaccines are produced through in vitro purification of target antigens and are relatively easy to manufacture. Compared to traditional vaccines, they do not contain genetic material and therefore offer a higher level of safety. As an important type of next-generation subunit vaccine, nanoparticle vaccines, whose scaffolds have plenty of independent surface-anchoring sites, can simultaneously display multiple antigenic components and possess advantages such as high safety, strong immunogenicity, and long-lasting immune responses [12].

They are considered a promising vaccine platform and have shown broad potential in the prevention and treatment of major diseases, including COVID-19, hepatitis B, influenza, AIDS, and cancer [13,14,15,16,17,18,19,20].

Nanoparticles can present antigens through several approaches, including genetic fusion, chemical conjugation, encapsulation, and embedding. Of these methods, genetic fusion (co-expression of antigen and nanoparticle scaffold protein genes) and non-fusion immobilization via SpyCatcher/SpyTag covalent anchoring have emerged as dominant research directions and are extensively adopted for veterinary vaccine development [21,22,23,24]. The SpyTag-SpyCatcher platform serves as a robust modular bioconjugation tool. Upon co-incubation under physiological environments, SpyCatcher specifically binds to SpyTag and mediates spontaneous formation of a covalent isopeptide bond without extra chemical catalysts. This feature renders rapid assembly of modular vaccines feasible.

Mammalian cell expression systems are widely used for the production of recombinant therapeutic proteins because of their ability to support accurate protein folding, assembly, and post-translational modifications [25]. Chinese hamster ovary (CHO) cells are among the most commonly used host cells in these systems. They support stable expression of exogenous proteins and possess a complete glycosylation machinery. Consequently, recombinant proteins produced in CHO cells closely resemble their native counterparts in terms of structure, characteristics, and biological function [26].

In this study, a recombinant ferritin nanoparticle vaccine based on the PEDV S1 domain was developed using a CHO cell expression system. After two immunizations, the immunogenicity and protective efficacy of the S1-Ferritin nanoparticle vaccine against PEDV were evaluated in mice and piglets. This work provides a promising platform for PEDV vaccine development and may facilitate the advancement of PEDV subunit vaccines.

## 2. Materials and Methods

### 2.1. Viruses and Cells

The S1 sequence was downloaded from NCBI under accession number KX534206, and PEDV JS-2/2015 was isolated and preserved by the Livestock Disease Research Laboratory of the Institute of Animal Science & Veterinary Medicine, Shanghai Academy of Agricultural Sciences. The ExpiCHO™ Expression System (Cat. no. A29133) was purchased from Invitrogen Trading (Shanghai) Co., Ltd. (Shanghai, China), a subsidiary of Life Technologies Corporation. After thawing, ExpiCHO-S cells were cultured in ExpiCHO™ Expression Medium according to the manufacturer’s instructions and used for transfection after three passages. The cells were maintained at 37 °C in an incubator with ≥80% relative humidity and 8% CO_2_ on a 19 mm orbital shaker at 125 rpm.

### 2.2. Design and Construction of Nanoparticle Vectors

The recombinant plasmid expressing ferritin was designed and constructed according to a previously reported method [13]. Briefly, I7E and N19Q mutations were introduced into Helicobacter pylori ferritin (GenBank NP_223316, residues 5-167) to eliminate the potential N-glycosylation site. The spycatcher gene was fused to the N-terminus of ferritin through a Ser-Gly-Gly linker and then cloned into the pET28a vector. All target genes were codon-optimized for *Escherichia coli* expression and synthesized by GenScript (Nanjing, China).

The N-terminal signal peptide of the PEDV S1 protein was deleted, and the spytag gene was fused to the C-terminus of S1 through a Ser-Gly-Gly linker. The codon-optimized S1-spytag (S1-spt) sequence for Homo sapiens was then cloned into the pcDNA3.1(+) plasmid with an N-terminal Kozak consensus sequence, a mouse Igκ signal peptide, and a C-terminal 8 × His tag. The construct was synthesized by GenScript.

### 2.3. Expression and Purification of Recombinant Ferritin and S1-spt

The recombinant plasmid pET28a-spycatcher-Ferritin (spc-Ferritin) was expressed in BL21 (DE3) *E. coli* and induced with IPTG at 37 °C for 6 h. Bacterial cells were collected by centrifugation, resuspended in lysis buffer (25 mM Tris-HCl, 150 mM NaCl, 1 mM PMSF, 100 μg/mL lysozyme, pH 8.5), and disrupted by high-pressure homogenization. The supernatant was then clarified by centrifugation. The spc-Ferritin nanoparticles were purified by ion-exchange chromatography using Q Sepharose Fast Flow resin (Tofflon Qianchun Biotechnology, Shanghai, China) after heat treatment at 70 °C for 15 min. Elution fractions were collected and analyzed by SDS-PAGE. The concentration of spc-Ferritin was determined using a BCA assay, and bacterial endotoxins were removed using a ToxinEraser™ Endotoxin Removal Kit (GenScript, Nanjing, China).

The S1-spt expression plasmid was transfected into CHO-S cells. Eight days later, the culture supernatant was collected and centrifuged to remove cellular debris. The supernatant was concentrated and filtered through a 0.45 μm membrane before purification. The clarified supernatant was loaded onto Ni-NTA resin (Tofflon Qianchun Biotechnology, Shanghai, China) to enrich His-tagged target proteins, followed by elution with imidazole-containing Tris buffer. The purified proteins were concentrated and exchanged into conventional Tris buffer. The concentration of S1-spt was determined by BCA assay, and the protein was stored at 4 °C for subsequent studies.

### 2.4. Generation and Purification of the S1-Ferritin Nanoparticle Protein

The spc-Ferritin and S1-spt proteins were incubated overnight at a molar ratio of 1:2 in 25 mM Tris, 150 mM NaCl (pH 7.5) at 4 °C. The resulting SpyTag/SpyCatcher-conjugated nanoparticle protein, S1-Ferritin, was purified by multimodal flow-through chromatography using a HiScale 26/20 column packed with Capto Core 700 resin (Cytiva, Marlborough, MA, USA). Elution fractions were collected over time. The target nanoparticles were recovered in the flow-through fraction, whereas excess S1-spt was retained by the multimodal chromatography medium. SDS-PAGE and Western blotting using an anti-His antibody (Cat. No. 66005 1 Ig, Proteintech, Wuhan, China) were performed to verify purity.

### 2.5. Transmission Electron Microscopy (TEM) and Dynamic Light Scattering (DLS) Analysis

The nanoparticles were further characterized by TEM at the Instrumental Analysis Center of Shanghai Jiao Tong University (Shanghai, China). Briefly, purified nanoparticles were applied to carbon-coated copper grids for 60 s. The samples were then negatively stained with phosphotungstic acid, and images were acquired using a Talos F200C G2 biological field-emission transmission electron microscope (FEI Company, Hillsboro, OR, USA ) operating at an accelerating voltage of 200 kV.

The hydrodynamic diameter (Z-average size) and time-dependent phase response of S1-Ferritin nanoparticles were measured using a Zetasizer Nano ZS instrument (Malvern Instruments Ltd., Worcestershire, UK). For DLS analysis, nanoparticles were diluted to 0.2 mg/mL in PBS, and the Z-average diameter was reported as nanometers (nm) ± distribution width. Phase signals (in radians) were recorded at 25 °C using the phase analysis module of the same instrument to monitor the dynamic response of nanoparticles under periodic excitation.

### 2.6. Vaccine Preparation

S1-Ferritin nanoparticles were administered at a dose of 20 μg protein in 0.2 mL for BALB/c mice and 200 μg protein in 2 mL for piglets. Based on preliminary dose-gradient assays, piglets received a 10-fold higher dose than mice after accounting for body weight differences and biosafety concerns. Montanide^TM^ ISA 201 adjuvant (Seppic, Paris, France) was mixed with the nanoparticle vaccine at a 1:1 ratio. The inactivated PEDV vaccine (IV) used in this study contained 10^7.5^ TCID50/mL of the PEDV JS-2/2015 strain. This viral concentration was confirmed based on pathogenic results from our team’s previous research during PEDV inactivated-vaccine application. The full-length trimeric S protein subunit vaccine of PEDV (S-Trimer) was produced using the insect baculovirus expression system and was provided by Yangling Kerry Biotechnology Co., Ltd. (Yangling, Shaanxi, China).

### 2.7. Immunization and Evaluation in Mice

Six-week-old female BALB/c mice (*n* = 30) were purchased from Shanghai JieSiJie Laboratory Animals Co., Ltd. (Shanghai, China) and randomly divided into five groups. Only female BALB/c mice were used in this study to avoid the influence of sex-related hormone differences and obtain more repeatable immune-related results. Mice were housed in a clean environment with free access to food and water. The indoor temperature was maintained at 22 °C ± 3 °C, the relative humidity was maintained at 50% ± 10%, and the animals were kept under a 12 h light/dark cycle.

To evaluate the immunogenicity of the S1-Ferritin nanoparticle vaccine, mice were primed subcutaneously with 20 μg of S1-Ferritin protein with or without ISA 201 adjuvant, 20 μg of S-Trimer subunit vaccine, 0.2 mL of inactivated PEDV vaccine (IV), or 0.2 mL of PBS. A booster immunization was administered 2 weeks after the primary immunization.

Serum and fecal samples were collected from all mice at the indicated time points after each immunization. Serum-specific IgG and IgG subclasses (IgG1 and IgG2a) in immunized mice were detected by enzyme-linked immunosorbent assay (ELISA) as previously described [27]. SIgA levels in fecal samples were measured using ELISA kits (Cat. No. SEKM-308; Solarbio, Beijing, China). Absorbance at 450 nm was measured using a Multiskan SkyHigh microplate spectrophotometer (A51119500C, Thermo Fisher Scientific, Waltham, MA, USA).

Serum levels of IFN-γ, IL-2, IL-4, and IL-12 P70 were also measured using commercial ELISA kits (IFN-γ, Cat. No. KE10094; IL-2, Cat. No. KE10126; IL-4, Cat. No. KE10010; IL-12 P70, Cat. No. KE10014; Proteintech, Wuhan, China). Both assays were performed using a double-antibody sandwich format. Briefly, microplates were pre-coated with purified anti-mouse antibodies specific for the target markers. After incubation with serum samples, HRP-conjugated detection antibodies were added to form immune complexes. Following color development with TMB substrate, the optical density (OD) was measured at 450 nm. Antibody and cytokine concentrations were calculated based on standard curves. All samples were analyzed in duplicate.

A virus neutralization assay was performed using PEDV JS-2/2015 to determine neutralizing antibodies (nAb) in mouse serum. Serum samples were heat-inactivated in a water bath at 56 °C for 30 min. All samples were tested in duplicate, starting at a dilution of 1:10, followed by two-fold serial dilutions, resulting in a total of 12 dilution levels. A volume of 100 μL was added to each well. An equal volume of virus suspension containing 200 TCID50 was mixed with the diluted serum and incubated at 37 °C for 1 h. The virus-serum mixtures were then added to vero cells and were allowed to adsorb for 1 h. Subsequently, the inoculum was removed and replaced with fresh medium containing 2% fetal bovine serum. Cells were cultured at 37 °C with 5% CO_2_ for 72 h and then examined for cytopathic effects (CPE). CPE-positive wells were recorded, and the 50% serum neutralization titer was calculated using the Reed–Muench method. Neutralizing titers were expressed as the reciprocal of the highest serum dilution that completely inhibited CPE formation.

### 2.8. T-Cell and B-Cell Activation Analysis by PCR Array

All mice were euthanized on day 28. Spleens were collected, homogenized, and centrifuged, and the resulting supernatants were harvested. Total RNA was extracted using a tissue and cellular RNA extraction kit based on the Trizol method (Cat. No. R30506, Yuanye Bio, Shanghai, China) according to the manufacturer’s instructions. RNA concentration and purity, represented by the A260/280 and A260/230 ratios, were measured using a NanoDrop spectrophotometer (Thermo Fisher Scientific). cDNA was synthesized from RNA using PrimeScript™ RT Master Mix (Cat. No. RR036A, Takara Bio, Shiga, Japan). T-Cell and B-Cell activation analysis was performed using a PCR array kit (Cat. No. WC-MRNA0138-M, WcGene Biotech, Shanghai, China). Each kit included validated internal reference controls (the complete gene list is presented in Table S1). Cycle threshold (Ct) values for each sample and target gene were obtained using a Bioer 9600 real-time PCR system (FQD-96A, Bioer, Hangzhou, China). Three internal reference genes (Actb, Gapdh and Hprt1) embedded within this kit were used, and the mean Ct value of the three housekeeping genes was used to normalize target-gene expression using the 2^−ΔΔCt^ method in accordance with the manufacturer’s recommendations.

### 2.9. Immunization and Evaluation of Piglets

A total of 12 conventionally weaned 10-day-old piglets were obtained from a commercial pig farm. All piglets were seronegative for PEDV antibodies (Keqian Biology, Wuhan, China) and tested negative for PEDV-N, PRRSV-ORF7, PCV2-cap and ASFV-B646L by real-time qPCR. Piglets were randomly assigned to three groups (*n* = 4 per group) and were provided feed and water ad libitum. The barn was cleaned twice daily, and manure was removed regularly.

The four piglets in the control group were further divided into two subgroups. Three piglets received an intramuscular injection of 2 mL PBS and were subsequently challenged with PEDV. The remaining piglet served as a negative control without immunization or viral challenge and was maintained under normal feeding conditions. Piglets in the IV group were immunized intramuscularly with 2 mL of inactivated PEDV vaccine. Piglets in the nanoparticle group received an intramuscular injection of 200 μg S1-Ferritin protein in 2 mL. All vaccinated piglets received a primary immunization on day 0 and a booster immunization on day 14. Serum samples were collected at 14 days after immunization for subsequent antibody analysis.

Detection of antigen-specific IgG and neutralizing antibodies in piglet sera was performed following the protocol described above. Serum concentrations of IFN-γ, IL-2, and IL-4 were determined using commercial ELISA kits (IFN-γ: ml002333; IL-2: ml025974; IL-4: ml025978; MlBio, Shanghai, China), according to the manufacturer’s instructions.

### 2.10. Piglet Challenge Test

Piglets in the immunized groups were orally challenged with 2 mL of 10^7.5^ TCID50 PEDV JS-2/2015 at 28 days after primary immunization. Following challenge, the mental status and clinical signs of the piglets were monitored daily. Fecal swabs were collected before challenge and daily thereafter for clinical scoring, and viral loads were quantified by real-time PCR. All piglets were humanely euthanized at 7 days post-challenge, and intestinal tissues were collected for histopathological analysis.

### 2.11. Histopathology

At necropsy, tissue samples from the mid-jejunum were collected. After fixation, tissues were dehydrated, cleared with xylene, embedded in paraffin, sectioned, and mounted on glass slides. Hematoxylin and eosin (H&E) staining and pathological examination were performed by the Laboratory Animal Center of Shanghai Jiao Tong University (Shanghai, China).

### 2.12. Statistical Analysis

GraphPad Prism 9.0 software was used for all statistical analyses. Data are presented as mean ± standard deviation (SD). Differences between two groups were analyzed using an unpaired *t*-test. *p*-values < 0.05 were considered statistically significant, whereas *p*-values < 0.01 were considered highly significant.

## 3. Results

### 3.1. Construction, Expression, and Purification of S1-Ferritin

**Figure 1:**
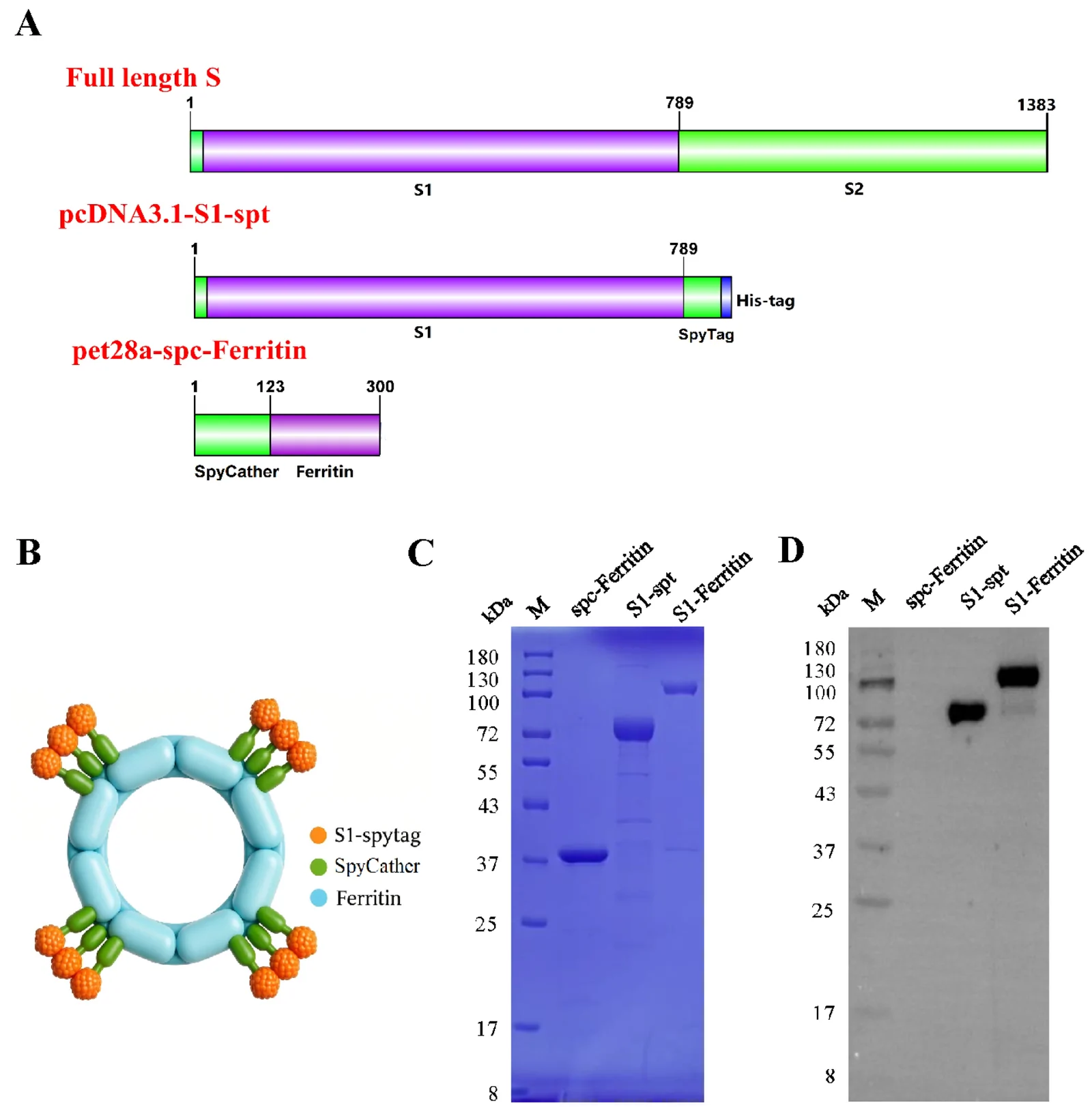
Analysis of the generation and expression characteristics of S1-Ferritin. (**A**) Schematic illustration of ferritin-based vaccine delivery vehicle. (**B**) Structural schematics of spc-Ferritin and S1-spt vector construction. (**C**) SDS-PAGE analysis of purified spc-Ferritin, S1-spt and S1-Ferritin nanoparticles. (**D**) Western blots of protein expression of spc-Ferritin, S1-spt and S1-Ferritin nanoparticles.

The S1-Ferritin nanoparticle was generated using a modified SpyTag/SpyCatcher system. A schematic diagram of the nanoparticle vaccine vector construction is shown in Figure 1A. The assembly strategy based on the S1 antigen, ferritin nano-scaffold, and SpyCatcher/SpyTag system is illustrated in Figure 1B. To obtain purified S1-Ferritin protein, CHO-S cells were transfected with the pcDNA3.1-S1-spt plasmid. After 8 days of expression, the culture supernatant was collected and purified using Capto Core 700 chromatography, while the ferritin nano-scaffold was purified from Escherichia coli using Q Sepharose Fast Flow resin. The target proteins were purified and characterized by SDS-PAGE and Western blotting. As shown in Figure 1C,D, both spc-Ferritin and S1-spt were successfully expressed. The S1-Ferritin nanoparticle protein generated through covalent coupling of spc-Ferritin and S1-spt at different molar ratios was successfully verified, with molecular weights of approximately 35, 65, and 100 kDa, respectively, which were consistent with the expected sizes.

### 3.2. Characterization of S1-Ferritin Nanoparticles

**Figure 2:**
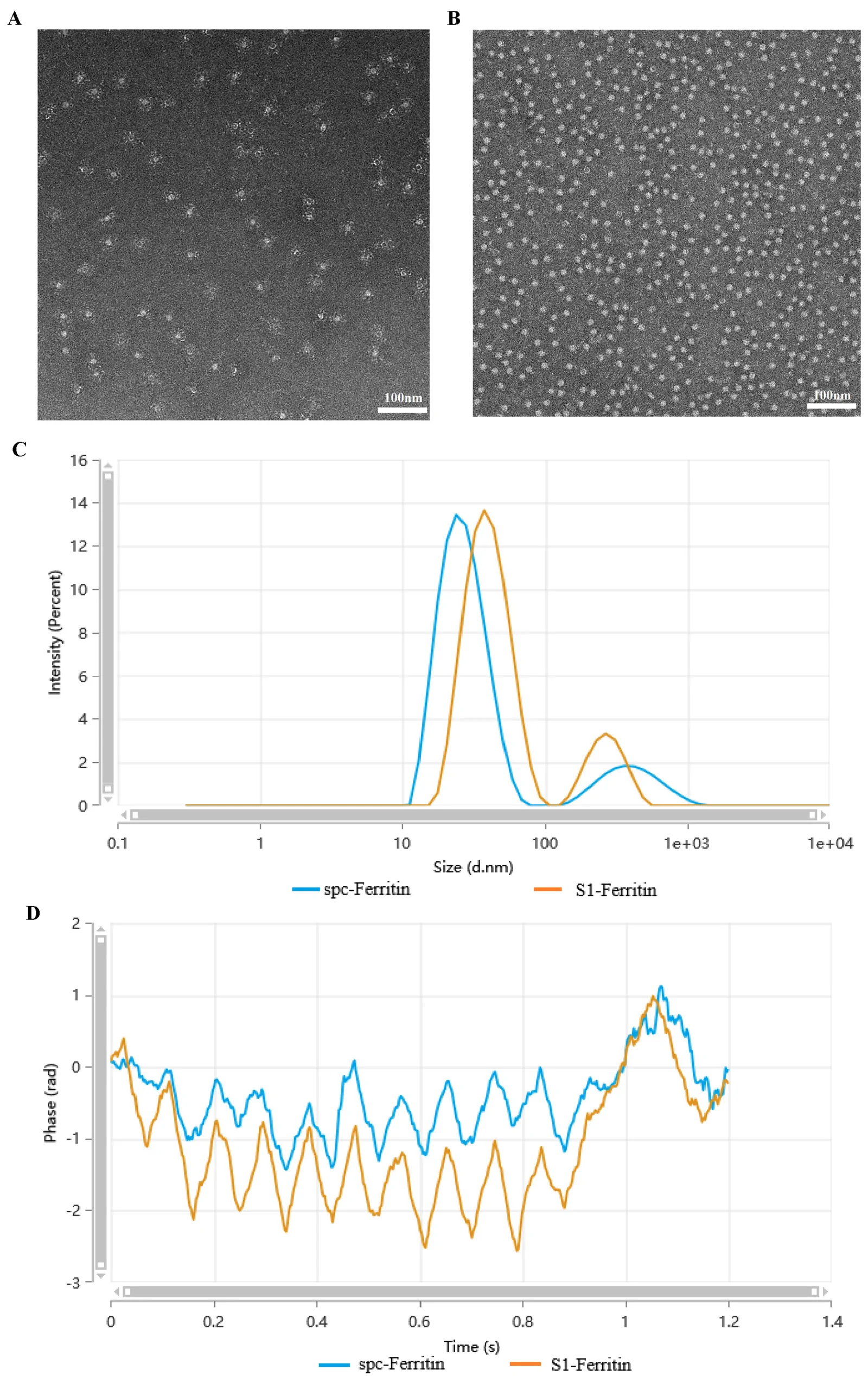
Morphology, particle size and phase response properties of S1-Ferritin nanoparticles. (**A**,**B**) TEM images showing nanoparticle morphology; (**C**) DLS measurement of particle size distribution (d.nm: diameter in nanometers); (**D**) Time-resolved phase response of S1-Ferritin nanoparticles (rad: radian).

To verify the self-assembly of the purified proteins into nanoparticles, transmission electron microscopy (TEM) and dynamic light scattering (DLS) were used to characterize their morphology and size. TEM analysis confirmed that both ferritin and S1-Ferritin self-assembled into highly uniform nanoparticles, with average diameters of approximately 15 nm and 40 nm, respectively (Figure 2A,B). Compared to the smooth and spherical morphology of ferritin nanoparticles, obvious protrusions were observed on the surface of S1-Ferritin nanoparticles, which were likely attributable to the displayed S1 proteins. DLS analysis showed that the Z-average diameter of S1-Ferritin nanoparticles was 43.53 nm (Figure 2C), which was consistent with the TEM results.

The phase-time profiles of S1-Ferritin (blue) and native spc-Ferritin (orange) showed distinct phase offsets and oscillation amplitudes (Figure 2D), indicating changes in particle properties after S1 modification.

### 3.3. S1-Ferritin Vaccination Induces Protective Immune Responses in BALB/c Mice

**Figure 3:**
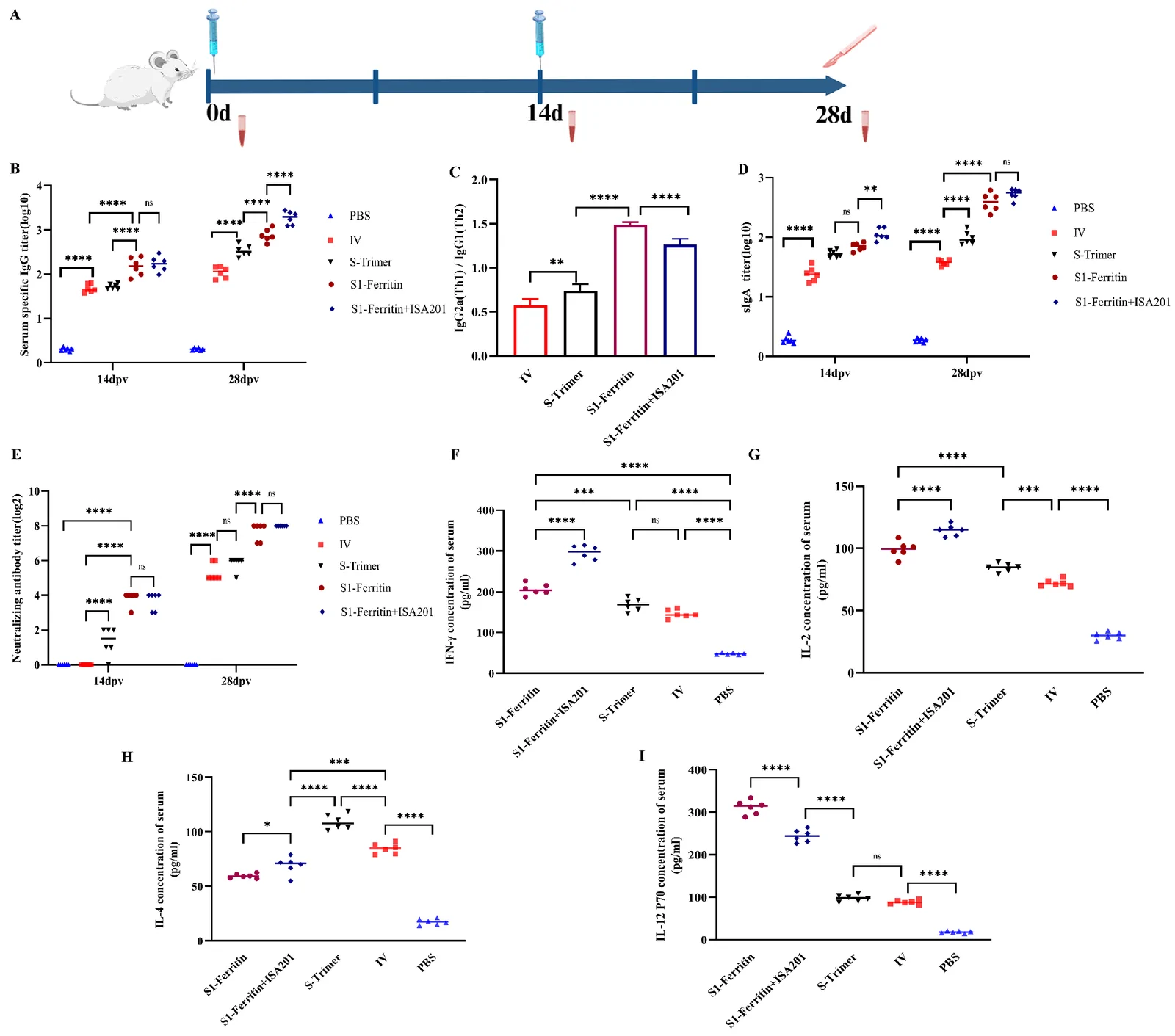
Protective immune responses elicited by the S1-Ferritin nanoparticle vaccine in mice. (**A**) Schematic illustration of immunization procedure and specimen collection in mice. (**B**) PEDV-specific serum IgG antibody titers determined on day 14 and day 28 post-immunization (*n* = 6) (dpv, days post-vaccination). (**C**) Analysis of serum IgG2a/IgG1 ratio to assess Th1/Th2 immune polarization following diverse vaccine inoculation. (**D**) Mucosal sIgA titers determined on day 14 and day 28 after vaccination. (**E**) PEDV-specific neutralizing antibody titers on day 14 and day 28 post-immunization. (**F**–**I**) Serum concentrations of IFN-γ, IL-2, IL-4 and IL-12 p70 from mice receiving different vaccine formulations 2 weeks after boost immunization. Individual data points with group median values are shown. * *p* < 0.05, ** *p* < 0.01, *** *p* < 0.001, **** *p* < 0.0001; ns, not significant.

Mouse immunization was performed according to the experimental schedule shown in Figure 3A. To evaluate the immunogenicity of S1-Ferritin nanoparticles, mice were immunized twice with S1-Ferritin nanoparticles formulated with or without ISA 201 adjuvant. The S-Trimer subunit vaccine and inactivated PEDV vaccine (IV) were used as vaccine controls, while PBS served as the negative control. The results showed that immunization with S1-Ferritin nanoparticles induced significantly higher IgG antibody responses than those observed in the S-Trimer and IV groups (Figure 3B). ISA 201 adjuvant further enhanced the IgG response induced by S1-Ferritin and generated markedly stronger IgG-associated antibody responses than the non-adjuvanted S1-Ferritin formulation. Analysis of the IgG2a/IgG1 ratio showed that the S1-Ferritin group exhibited a relatively higher IgG2a response than the control groups, suggesting a Th1-oriented immune response. In contrast, the ISA 201-adjuvanted S1-Ferritin group tended to induce a more balanced immune response, whereas the S-Trimer and IV groups mainly triggered a Th2-biased response (Figure 3C).

SIgA is the predominant antibody isotype in mucosal surfaces and serves as an important first line of defense against pathogen invasion. To evaluate the mucosal immune response induced by the S1-Ferritin nanoparticle vaccine, sIgA levels in fecal samples were measured by ELISA after immunization. As shown in Figure 3D, mice vaccinated with the S1-Ferritin nanoparticle vaccine produced significantly higher levels of sIgA than those in the control groups. Notably, ISA 201 adjuvant further increased sIgA production on 14 days after primary immunization, although no significant difference was observed at day 28. These findings suggest that the S1-Ferritin nanoparticle vaccine can induce a strong mucosal immune response, which is important for preventing PEDV infection.

Neutralizing antibodies (NAbs) play a key role in protection against PEDV infection and are an important indicator for evaluating vaccine efficacy. The kinetics of PEDV JS-2/2015-specific NAb responses induced by the S1-Ferritin nanoparticle vaccine followed a pattern similar to that observed for antigen-specific IgG responses. However, no significant difference was detected between the S1-Ferritin group and the ISA 201-adjuvanted S1-Ferritin group (Figure 3E). At day 28, the S1-Ferritin vaccine induced a peak NAb titer of log_2_ 8, whereas the commercial inactivated vaccine (IV) induced a titer of only log_2_ 6. Interestingly, the addition of ISA 201 adjuvant did not further enhance the neutralizing antibody response induced by the S1-Ferritin nanoparticle vaccine, suggesting that the adjuvant did not provide additional improvement in functional humoral immunity under the conditions tested.

Cytokines play important roles in regulating innate immunity and shaping the magnitude and polarization of adaptive immune responses. Serum cytokine levels were measured by ELISA at 2 weeks after booster immunization. As shown in Figure 3F–I, both IFN-γ and IL-2 levels were significantly higher in mice vaccinated with S1-Ferritin or ISA 201-adjuvanted S1-Ferritin than in mice receiving the S-Trimer or inactivated vaccine. For IL-12 p70, the S1-Ferritin group showed the highest cytokine level, which was significantly higher than that observed in the ISA 201-adjuvanted S1-Ferritin group. In contrast, higher IL-4 production was detected in the S-Trimer and IV groups than in the nanoparticle vaccine groups, further supporting a tendency toward Th2-type immune responses in these groups.

Taken together, these findings demonstrate that the S1-Ferritin nanoparticle vaccine can induce high levels of antibody and cytokine responses, highlighting its potential as a promising vaccine candidate against PEDV.

### 3.4. T-Cell and B-Cell Activation Analysis

T- and B-cell activation is a key step in the initiation of adaptive immunity and plays an important role in triggering antigen-specific cellular and humoral immune responses, thereby influencing the protective efficacy of vaccine candidates. A PCR array was used to evaluate the expression of genes associated with T- and B-cell activation in mouse spleen tissues.

**Figure 4:**
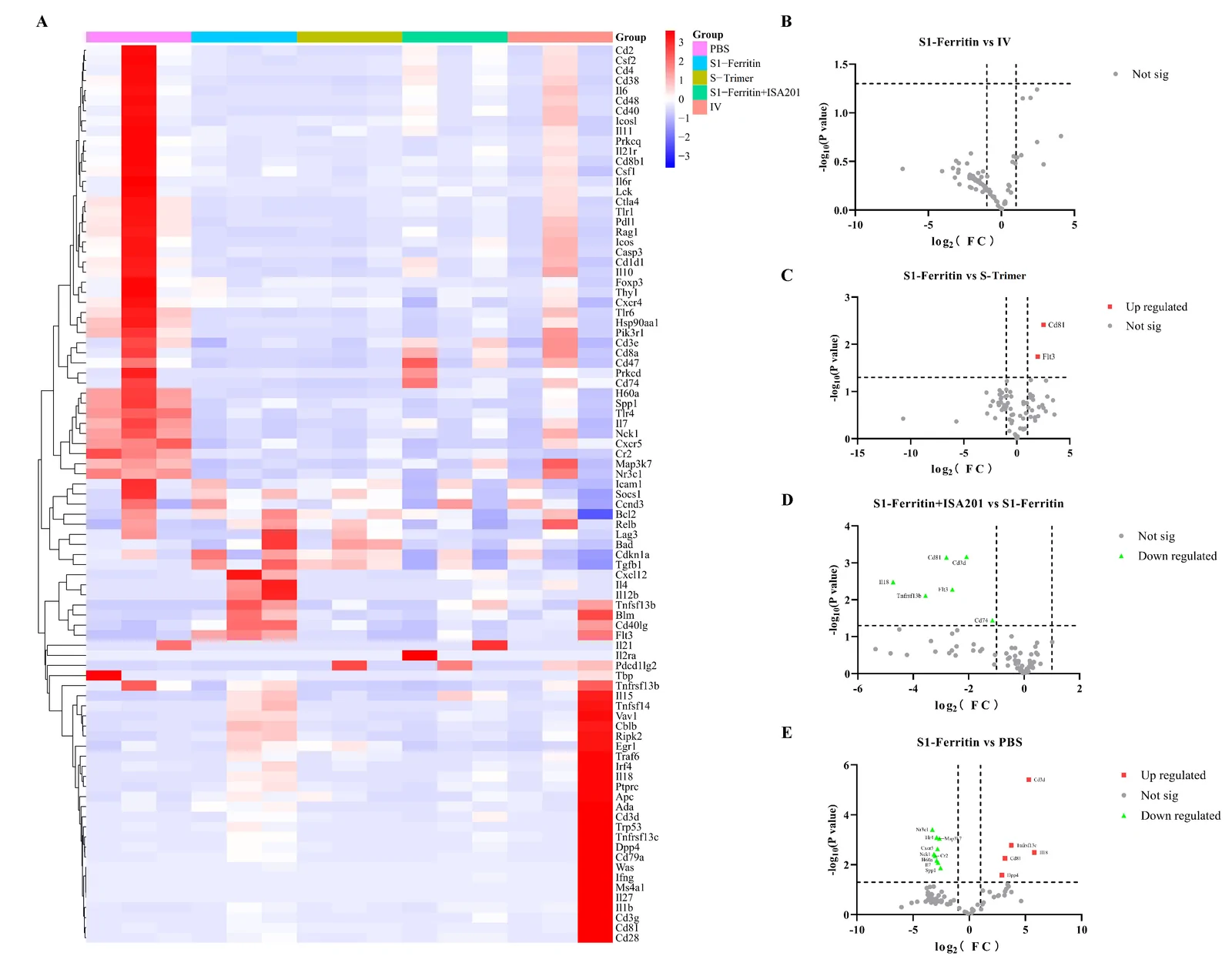
Gene expression profiles detected by PCR Array in spleen tissues of mice with different treatments. (**A**) Heatmap showing normalized expression levels of target genes across all experimental groups. (**B**–**E**) Volcano plot of differentially expressed genes between the S1-Ferritin and other groups (Not sig, not significant).

As shown in the heatmap (Figure 4A) and volcano plots (Figure 4B–E), comparison between the S1-Ferritin and PBS groups revealed that the S1-Ferritin nanoparticle vaccine markedly altered the expression of multiple genes involved in splenic T- and B-cell activation. Several key genes were significantly upregulated, including the T-cell marker Cd3d, which encodes the CD3δ chain and serves as an important indicator of T-cell activation, and the B-cell activation molecule Cd81, which encodes a tetraspanin protein that enhances BCR signaling and promotes B-cell activation. Consistent with this observation, comparison with the S-Trimer group showed that the S1-Ferritin vaccine significantly increased the expression of two important immune-related genes, Cd81 and Flt3, further supporting its ability to promote lymphocyte activation. In contrast, no obvious differentially expressed genes were detected between the IV and S1-Ferritin groups, suggesting that the two treatments induced highly similar gene expression profiles associated with splenic T- and B-cell activation. Notably, compared with the S1-Ferritin group, the ISA201-adjuvanted S1-Ferritin group exhibited six significantly downregulated genes, including Cd3d, Cd81, Flt3, Il18, Tnfrsf13b, and Cd74, while no significantly upregulated genes were detected. These results suggest that ISA201 did not enhance the expression of key T- and B-cell activation genes and may instead have suppressed their expression under the conditions tested.

For the heatmap: Each row represents an individual gene, and each column represents a mouse spleen sample from one of the five groups (PBS, S1-Ferritin, S-Trimer, S1-Ferritin + ISA 201, and IV). Relative gene expression levels were normalized using Z-scores. Red indicates high expression, whereas blue indicates low expression. Hierarchical clustering was performed based on gene expression patterns (*n* = 3).

For all volcano plots: The x-axis represents log_2_-transformed fold change (log_2_FC) in gene expression, and the y-axis represents −log_10_-transformed *p*-values. Genes with log_2_FC ≥ 1 and *p* < 0.05 were considered significantly differentially expressed. The horizontal dashed line indicates *p* = 0.05, and the vertical dashed lines indicate log_2_FC = ±1. Gray dots represent genes with no significant changes in expression. Red squares and green triangles indicate significantly upregulated and downregulated genes, respectively. Representative differentially expressed genes are labeled in each plot.

### 3.5. S1-Ferritin Elicits Protective Immune Responses in Pigs

**Figure 5:**
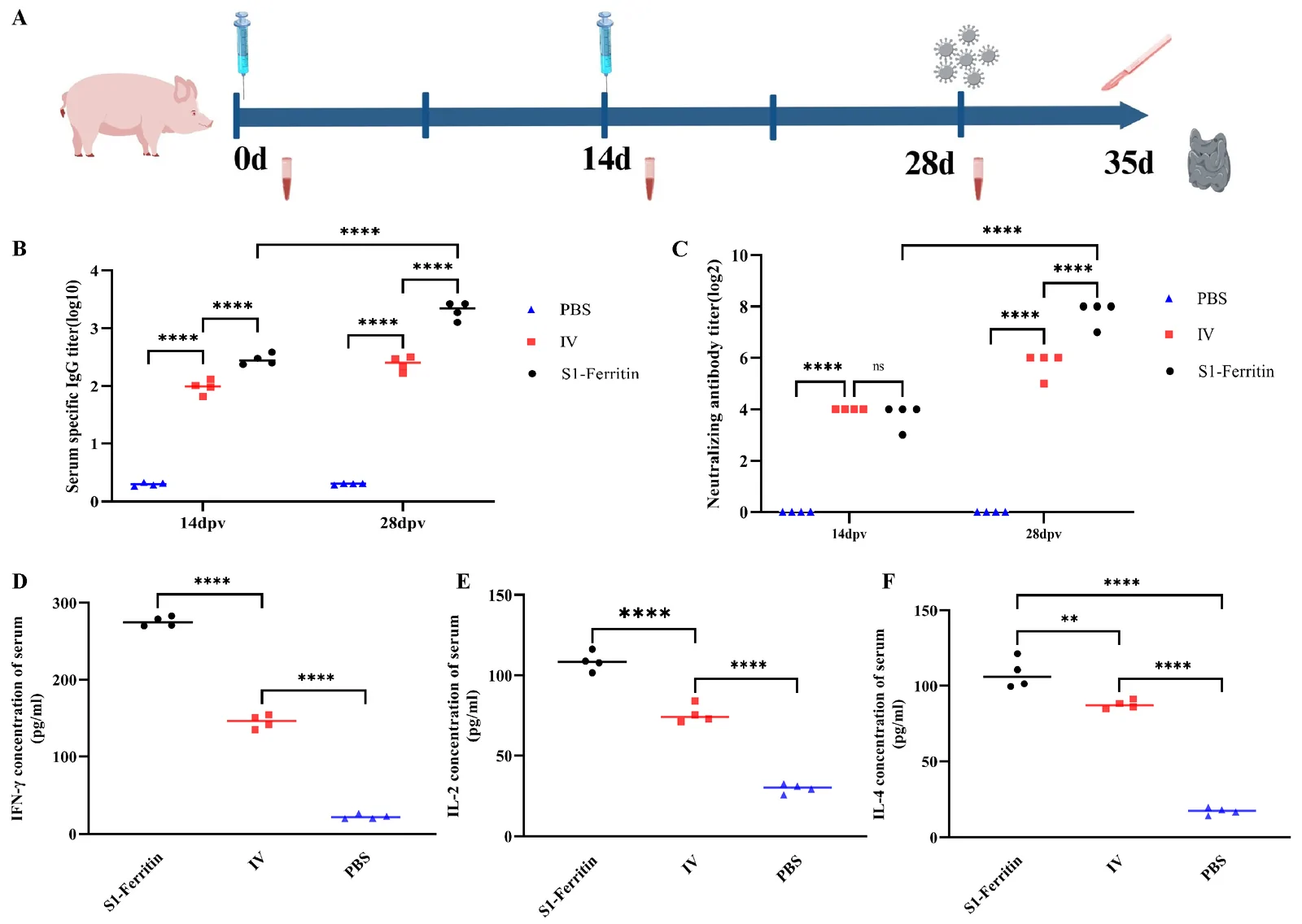
Protective immune responses induced by the S1-Ferritin nanoparticle vaccine in piglets. (**A**) Schematic timeline of piglet immunization and sample collection. (**B**) PEDV S1-specific serum IgG titers measured on 14 and 28 days post-immunization (n = 4). (**C**) Serum neutralizing antibody titers determined on day 14 and day 28 post-immunization. (**D**–**F**) Serum levels of IFN-γ, IL-2 and IL-4 sampled at two weeks post-booster vaccination. Individual data and group median values are presented. ** *p* < 0.01, **** *p* < 0.0001; ns, no significant difference.

To assess the protective immune responses induced by different vaccine formulations, piglets were randomly assigned to receive the S1-Ferritin nanoparticle vaccine, inactivated vaccine, or PBS. The immunization and challenge schedule used in piglets is shown in Figure 5A. Briefly, piglets were vaccinated twice at weeks 0 and 2. Serum antigen-specific IgG and neutralizing antibodies were detected, and serum cytokines were measured by ELISA. Two weeks after booster immunization, piglets were orally challenged with the PEDV JS-2/2015 strain. Rectal temperature, viral loads in rectal swabs, and gross lesions in the small intestine were subsequently evaluated.

Measurement of S1-specific IgG in piglets confirmed strong humoral immune responses in both the S1-Ferritin and inactivated PEDV (IV) vaccine groups. The S1-Ferritin group generated significantly higher serum IgG titers than the IV group at both 14 and 28 days after immunization. Neutralizing antibody levels were similar between the two vaccine groups on day 14. However, S1-Ferritin induced significantly stronger neutralizing activity at day 28 after booster immunization. For cellular immune responses, serum levels of IFN-γ, IL-2 (Th1), and IL-4 (Th2) were markedly higher in both vaccine groups than in the PBS control group. Moreover, S1-Ferritin vaccination induced higher levels of all three cytokines than IV vaccination.

Taken together, the results obtained in piglets demonstrate that the S1-Ferritin nanoparticle vaccine enhances the immunogenicity of PEDV S1 by inducing strong humoral immune responses and balanced Th1/Th2 immunity.

### 3.6. S1-Ferritin Alleviates Intestinal Pathological Lesions Following PEDV Challenge

At 28 days post-immunization, all piglets were orally challenged with 2 mL of PEDV JS-2/2015 at a dose of 10^7.5^ TCID50. Following challenge, piglets were monitored daily for 7 consecutive days to assess general condition, feed intake, and diarrhea occurrence. Rectal swabs were collected daily from each piglet, and PEDV shedding was measured by qRT-PCR. All surviving piglets were humanely euthanized at 7 days post-challenge, and jejunal tissues were collected for histopathological analysis.

Both the S1-Ferritin nanoparticle vaccine and the inactivated vaccine provided complete clinical protection against PEDV challenge in piglets. Following challenge, no obvious clinical symptoms were observed in either vaccinated group. In contrast, piglets in the PBS-challenged group developed persistent fever ranging from 38.9 to 40.1 °C, accompanied by diarrhea, depression, and reduced feed intake.

**Figure 6:**
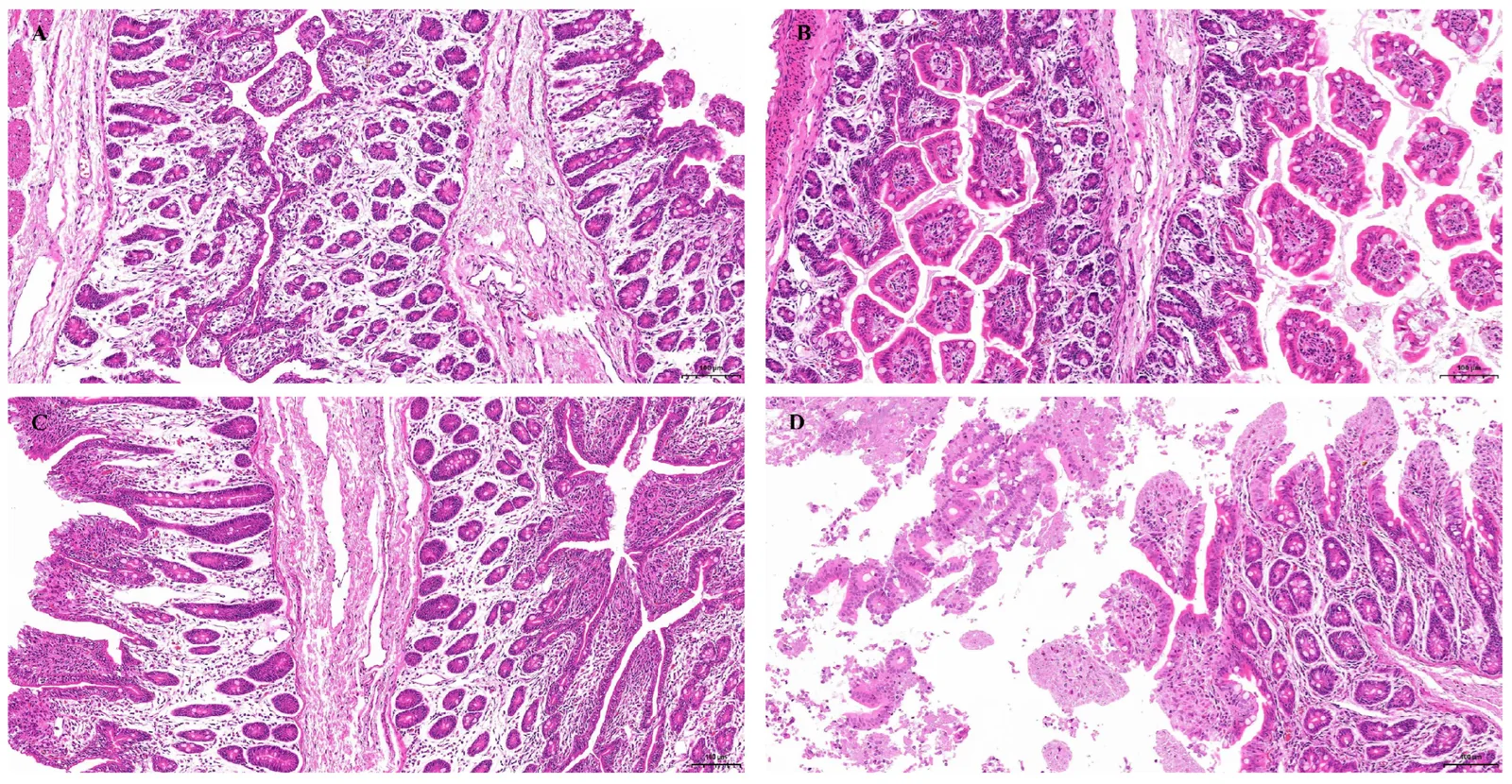
Histopathological analysis of small intestinal pathological lesions in piglets following PEDV challenge. (**A**–**D**): Representative H&E-stained small intestinal sections from experimental groups. (**A**): S1-Ferritin nanoparticle vaccine group. (**B**): Inactivated PEDV vaccine group. (**C**): Negative control. (**D**): PBS-challenged group. (Scale bar = 100 µm).

Histopathological examination of jejunal tissues by HE staining revealed severe lesions in piglets from the PBS-challenged group (Figure 6D). These lesions included marked villous blunting, villous atrophy, epithelial exfoliation, and extensive inflammatory cell infiltration within the lamina propria. In contrast, piglets vaccinated with either the S1-Ferritin nanoparticle vaccine or the inactivated vaccine, as well as those in the PBS negative control group, displayed intact intestinal villi, well-organized mucosal architecture, and no obvious inflammatory lesions in the jejunum(Figure 6A–C).

Monitoring of PEDV shedding showed that sustained viral excretion occurred in the PBS-challenged control group from day 3 to day 7 post-challenge, with a peak viral load reaching 4.1 × 10^7^ copies/mL. In contrast, PEDV was not detected in rectal swab samples collected from piglets vaccinated with either the S1-Ferritin nanoparticle vaccine or the inactivated vaccine. These findings indicate that the S1-Ferritin nanoparticle vaccine can effectively suppress PEDV replication in vivo and reduce viral shedding.

## 4. Discussion

The continuous circulation of mutated PEDV variants has increasingly challenged the protective efficacy of traditional vaccines. As versatile and promising vaccine delivery platforms, nanoparticles have shown considerable potential for preventing a wide range of infectious diseases [13,28,29,30,31], from coronaviruses to influenza viruses, making them attractive candidates for the development of next-generation PEDV vaccines.

Biologically self-assembled proteins, including ferritin, Lumazine synthase (LS), bacteriophage coat protein AP205, and mutated-i301 scaffold protein, have been widely studied as antigen display platforms for swine enteric viruses [32,33,34,35,36,37]. Yang [32] developed self-assembled nanoparticle vaccines targeting the N-terminal domain (NTD) and C-terminal domain (CTD) of the PEDV S1 protein. Both vaccines induced high levels of neutralizing antibodies, and a 3:1 combination of the two formulations provided strong protection against PEDV challenge in piglets. Kim and co-workers [33] constructed a ferritin nanoparticle conjugate vaccine fused with PEDV RBD-specific affinity antibody Fv fragments, which showed potent neutralizing activity in immunized mice. The team of the Lanzhou Veterinary Research Institute [34] developed an S1-trimer-displayed nanoparticle vaccine that induced strong neutralizing antibody responses, reduced tissue damage, and decreased viral loads in vaccinated piglets. Gu [35] engineered an AP205-S1 nanoparticle vaccine candidate targeting PEDV G-II strains, which induced satisfactory immunogenicity and cross-neutralizing activity against G-I strains in mice and provided effective protection against PEDV infection in piglets. Li [36] et al. constructed a PEDV nanoparticle vaccine based on Mi3 nanoparticles. Immunization of mice induced robust PEDV-specific IgG, sIgA, neutralizing antibody, and cytokine responses, demonstrating good immunogenicity. A ferritin nanoparticle vaccine carrying the PDCoV RBD was also developed by the Shanghai Veterinary Research Institute [37]. Strong antigen-specific IgG and neutralizing antibody responses were observed in mice, together with effective protection against PDCoV challenge. In the present study, we successfully developed an S1-Ferritin nanoparticle vaccine using the SpyTag/SpyCatcher modular conjugation system and systematically evaluated its protective efficacy against PEDV challenge in piglets. Consistent with previous studies on PEDV nanoparticle vaccines, our results showed that the nanoparticle vaccine induced strong humoral and cellular immune responses in mice and provided effective protection against PEDV infection in piglets. These findings provide further support for the application of nanoparticle-based vaccines in the prevention and control of swine diarrheal diseases.

Mucosal immunity represents the first barrier against viral invasion [38]. By mimicking the size and structural features of natural pathogens, nanoparticle vaccines can effectively stimulate mucosal immune responses and provide cross-protection. In this study, high levels of secretory immunoglobulin A (sIgA) were detected in fecal samples from mice immunized with the nanoparticle vaccine, which is consistent with findings reported in a previous study [36]. Additionally, PEDV mainly causes enteric disease characterized by diarrhea, and elevated intestinal sIgA levels play an important role in reducing intestinal injury and alleviating diarrheal symptoms.

Cytokines play an important role in immune regulation and anti-infection. In the present study, S1-Ferritin nanoparticle vaccine markedly increased the production of IL-2, IL-4, IL-12 P70 and IFN-γ, supporting robust systemic and mucosal immunity. IL-2, IL-12 P70 and IFN-γ drive Th1-dominated cellular immunity by activating T-cell and NK-cell responses, whereas IL-4 initiates Th2-type responses for persistent antibody generation and mucosal memory. Conventional inactivated vaccines usually induce limited cytokine release and skewed Th2-biased immunity. By enhancing antigen presentation and extending antigen retention, the S1-Ferritin nanoparticle vaccine induces well-balanced Th1/Th2 immune responses, which is critical for the prevention and control of PEDV infection.

Ferritin is a well-characterized self-assembling protein scaffold with excellent biocompatibility and a highly uniform nanostructure [39]. These properties allow multivalent presentation of viral antigens and improve antigen presentation efficiency. Compared with soluble S-Trimer protein, the S1-Ferritin nanoparticle can effectively concentrate S1 epitopes on the particle surface, thereby overcoming the relatively low immunogenicity of soluble subunit antigens. Meanwhile, the SpyTag/SpyCatcher coupling system used in this study enables rapid, specific, and covalent assembly of antigens without the need for complex genetic engineering, providing flexibility for the rapid development of vaccines against newly emerging PEDV variants. Compared to conventional recombinant or inactivated vaccines, this modular assembly strategy can considerably shorten vaccine development and production timelines. Previous studies have also demonstrated the feasibility of this modular vaccine strategy in the prevention and control of a variety of animal diseases [22,24,32].

Nevertheless, this study still has several limitations. First, all experiments were conducted under controlled laboratory conditions. Further evaluation in commercial pig farms with more complex pathogen exposure and different maternal antibody backgrounds is needed to better assess vaccine performance under field conditions. Second, the long-term durability of the immune response remains to be investigated, and the cross-protective efficacy against genetically diverse PEDV variants requires further validation. Third, the precise molecular mechanisms through which the nanoparticle scaffold regulates dendritic cell activation and T-/B-cell differentiation have yet to be fully clarified.

Future studies will include large-scale field trials in commercial pig farms. In addition to screening proper doses and vaccination schemes, oral and intranasal immunization will be implemented to assess the mucosal immune potential of our nanoparticle vaccine, followed by long-term surveillance of antibody responses and clinical outcomes upon natural PEDV challenge.

## 5. Conclusions

In conclusion, our work confirms that SpyTag-SpyCatcher-assembled S1-Ferritin nanoparticles are capable of inducing strong, comprehensive immune responses in both mice and piglets and constitute a safe, stable and effective vaccine candidate to prevent PEDV. Such a modular nanoparticle platform affords a practical and universally applicable technical solution for rapid development and performance upgrading of next-generation swine antiviral vaccines.

## Data Availability

The data presented in this study are available upon request from the corresponding authors. The data are not publicly available due to privacy concerns.

## Supplementary Materials

The following supporting information can be downloaded at: https://www.mdpi.com/article/10.3390/biom16081090/s1, Figure S1: SDS-PAGE analysis of purified spc-Ferritin, S1-spt and S1-Ferritin nanoparticles; Figure S2: Western blots of protein expression of spc-Ferritin, S1-spt and S1-Ferritin nanoparticles; Table S1: Gene List for T-Cell and B-Cell Activation PCR Array plate. PDF S1: Academic Manuscript Editing Certificate.
