# Supplementary material for: Nanoparticle Vaccine Based on S1 Domain of Porcine Epidemic Diarrhea Virus Elicits Protective Immune Responses in Mice and Pigs
Source: Biomolecules. 2026 Jul 25;16(8):1090. doi: 10.3390/biom16081090 (PMC13511541; doi:10.3390/biom16081090)
Supplement: Supplementary file 1 [file biomolecules-16-01090-s001.zip › Figures S1 and S2.pdf]

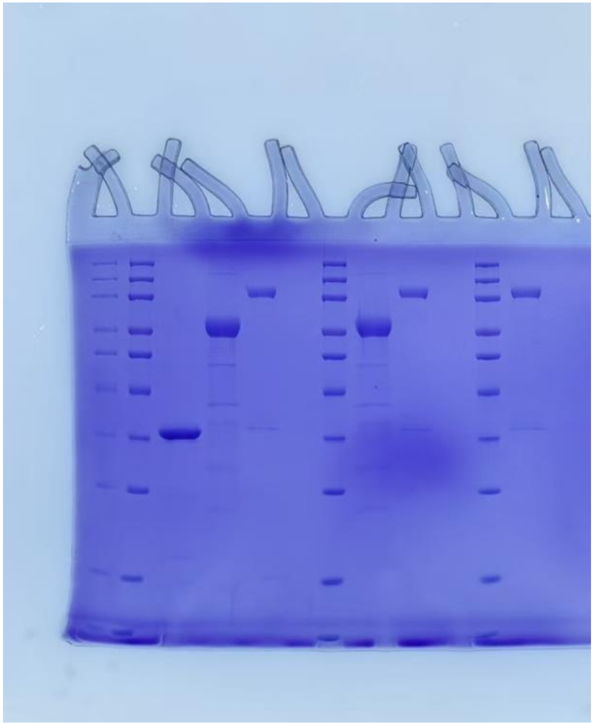

Figure S1. SDS-PAGE analysis of purified spc-Ferritin, S1-spt and S1-Ferritin nanoparticles.

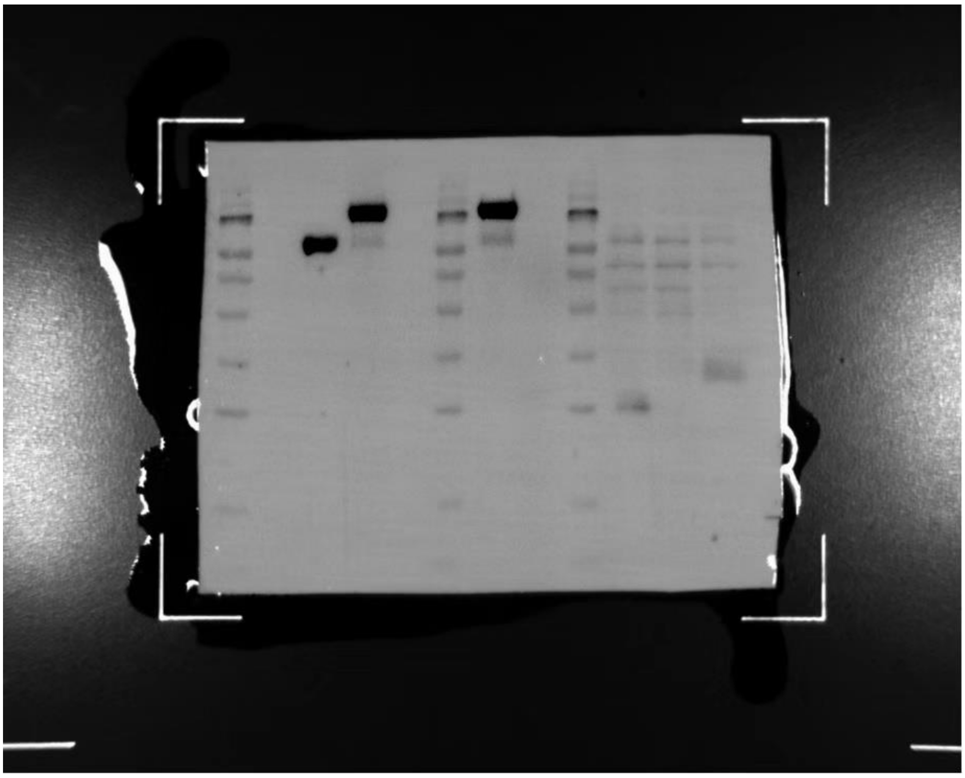

Figure S2. Western blots of protein expression of spc-Ferritin, S1-spt and S1-Ferritin nanoparticles.
