# Supplementary material for: Nanoparticle Vaccine Based on S1 Domain of Porcine Epidemic Diarrhea Virus Elicits Protective Immune Responses in Mice and Pigs
Source: Biomolecules. 2026 Jul 25;16(8):1090. doi: 10.3390/biom16081090 (PMC13511541; doi:10.3390/biom16081090)
Supplement: Supplementary file 1 [file biomolecules-16-01090-s001.zip › PDF S1.pdf]

EDITORIAL CERTIFICATE

This is to certify that the manuscript detailed below was edited by one or more of our highly qualified, native English speakers at EditSprings, to assure compliance with Anglophone academic standards in terms of style, punctuation, grammar, and spelling.

Manuscript title:

Nanoparticle vaccine based on S1 domain of porcine epidemic diarrhea virus elicits protective immune responses in mice and pigs

Authors:

Pan Tang , Benqiang Li ,Enhui Cui , Jie Tao , Jinghua Cheng , Ying Shi , Li Qi, Lv Lilei, Huili Liu *

Date Issued:

June 21, 2026

Certificate Number:

ES-202606081224622653

This certificate can be verified on https://www.editsprings.com/QueryCertificate.html. EditSprings hereby certifies that neither content nor the author's intentions were altered in any way during the editing process. Documents in receipt of this certification should be ready for publication as far as style and language are concerned, provided that the author(s) accepted our suggestions and changes (which remains their right and responsibility).

EditSprings offers a wide range of editing, translation, for researchers and publishers across the world. Our highly skilled editors are all established academics based in Anglophone Higher Education institutions across the world (U.K., U.S.A., Canada, Australia, and elsewhere), are experts in their respective fields, and are qualified to edit research papers authored by non-Anglophone scholars.
