# Supplementary material for: Nanoparticle Vaccine Based on S1 Domain of Porcine Epidemic Diarrhea Virus Elicits Protective Immune Responses in Mice and Pigs
Source: Biomolecules. 2026 Jul 25;16(8):1090. doi: 10.3390/biom16081090 (PMC13511541; doi:10.3390/biom16081090)
Supplement: Supplementary file 1 [file biomolecules-16-01090-s001.zip › Table S1.pdf]

Table S1:

T-Cell and B-Cell Activation PCR Array plate

Gene List for Kit Detection:

mouse	1	2	3	4	5	6	7	8	9	10	11
A	Ada	Apc	Bad	Bcl2	Blm	Casp3	Cblb	Ccnd3	Cd1d1	Cd2	Cd28
B	Cd3d	Cd3e	Cd3g	Cd4	Cd40	Cd40lg	Cd47	Cd48	Cd74	Cd79a	Cd81
C	Cd8b1	Cdkn1a	Cr2	Csf1	Csf2	Ctla4	Cxcl12	Cxcr4	Cxcr5	Dpp4	Egr1
D	Foxp3	H60a	Hsp90aa1	Icam1	Icos	Icosl	Ifng	Il10	Il11	Il12b	Il15
E	Il1b	Il21	Il21r	Il27	Il2ra	Il4	Il6	Il6r	Il7	Irf4	Lag3
F	Map3k7	Ms4a1	Nck1	Nos2	Nr3c1	Pdcd1lg2	Pdl1	Pik3r1	Prkcd	Prkcq	Ptprc
G	Relb	Ripk2	Socs1	Spp1	Tbp	Tgfb1	Thy1	Tlr1	Tlr4	Tlr6	Tnfrsf13b

Name	Description
Ada	adenosine deaminase
Cd3d	CD3 antigen, delta polypeptide
Cd8b1	CD8 subunit beta 1
Foxp3	forkhead box P3
Il1b	interleukin 1 beta
Map3k7	mitogen-activated protein kinase kinase kinase 7
Relb	Relb proto-oncogene, NFKB subunit
Apc	APC, WNT signaling pathway regulator
Cd3e	CD3 antigen, epsilon polypeptide
Cdkn1a	cyclin dependent kinase inhibitor 1A
H60a	histocompatibility 60a
Il21	interleukin 21
Ms4a1	membrane-spanning 4-domains, subfamily A, member 1
Ripk2	receptor (TNFRSF)-interacting serine-threonine kinase 2
Bad	BCL2-associated agonist of cell death
Cd3g	CD3 antigen, gamma polypeptide
Cr2	complement receptor 2
Hsp90aa1	heat shock protein 90, alpha (cytosolic), class A member 1
Il21r	interleukin 21 receptor
Nck1	non-catalytic region of tyrosine kinase adaptor protein 1
Socs1	suppressor of cytokine signaling 1
Bcl2	B cell leukemia/lymphoma 2
Cd4	CD4 antigen
Csf1	colony stimulating factor 1 (macrophage)
Icam1	intercellular adhesion molecule 1

Il27	interleukin 27
Nos2	nitric oxide synthase 2, inducible
Spp1	secreted phosphoprotein 1
Blm	Bloom syndrome, RecQ like helicase
Cd40	CD40 antigen
Csf2	colony stimulating factor 2 (granulocyte-macrophage)
Icos	inducible T cell co-stimulator
Il2ra	interleukin 2 receptor, alpha chain
Nr3c1	nuclear receptor subfamily 3, group C, member 1
Tbp	TATA box binding protein
Casp3	caspase 3
Cd40lg	CD40 ligand
Ctla4	cytotoxic T-lymphocyte-associated protein 4
Icosl	icos ligand
Il4	interleukin 4
Pdcd1lg2	programmed cell death 1 ligand 2
Tgfb1	transforming growth factor, beta 1
Cblb	Cbl proto-oncogene b
Cd47	CD47 antigen (Rh-related antigen, integrin-associated signal transducer)
Cxcl12	C-X-C motif chemokine ligand 12
Ifng	interferon gamma
Il6	interleukin 6
Pdl1	CD274 antigen
Thy1	thymus cell antigen 1, theta
Ccnd3	cyclin D3

Cd48	CD48 antigen
Cxcr4	C-X-C motif chemokine receptor 4
Il10	interleukin 10
Il6r	interleukin 6 receptor, alpha
Pik3r1	phosphoinositide-3-kinase regulatory subunit 1
Tlr1	toll-like receptor 1
Cd1d1	CD1d1 antigen
Cd74	CD74 antigen (invariant polypeptide of major histocompatibility complex, class II antigen-associated)
Cxcr5	C-X-C motif chemokine receptor 5
Il11	interleukin 11
Il7	interleukin 7
Prkcd	protein kinase C, delta
Tlr4	toll-like receptor 4
Cd2	CD2 antigen
Cd79a	CD79A antigen (immunoglobulin-associated alpha)
Dpp4	dipeptidylpeptidase 4
Il12b	interleukin 12b
Irf4	interferon regulatory factor 4
Prkcq	protein kinase C, theta
Tlr6	toll-like receptor 6
Cd28	CD28 antigen
Cd81	CD81 antigen
Egr1	early growth response 1
Il15	interleukin 15
Lag3	lymphocyte-activation gene 3

Ptprc	protein tyrosine phosphatase receptor type C
Tnfrsf13b	tumor necrosis factor receptor superfamily, member 13b
